# Lithiasis of Minor Salivary Gland Mimicking a Venous Malformation: Contextual Diode Laser Photocoagulation and Excision

**DOI:** 10.7759/cureus.7300

**Published:** 2020-03-17

**Authors:** Giuseppe Barile, Eleonora Quercia, Leonardo Barulli, Eugenio Maiorano

**Affiliations:** 1 Department of Interdisciplinary Medicine, University of Bari Aldo Moro, Bari, ITA; 2 Emergency and Organ Transplantation, University of Bari Aldo Moro, Bari, ITA

**Keywords:** lithiasis, minor salivary gland, venous malformation, diode laser, photocoagulation, cheek

## Abstract

Sialolithiasis is the most common disease of the major salivary gland. Lithiasis of minor salivary gland, instead, is very rare. Diagnosis is often challenging as frequency, clinical appearance and symptoms are relatively unknown. We report on a case of minor salivary gland lithiasis of the cheek resembling a venous malformation and its management by diode laser.

## Introduction

Minor salivary gland lithiasis (MSGL) is extremely rare, as it represents approximately two percent of all cases of sialolithiasis [1, 2]. Although data reported in the literature on frequency, signs, and symptoms of MSGL are relatively unknown, it is generally described as a firm, solitary, submucosal painless nodule with hard consistency and mobility in the surrounding tissue, mainly occurring in lips and buccal mucosa [1-4]. MSGL is frequently misdiagnosed, especially when associated with masticatory trauma and/or resembling other benign minor salivary gland disease [1, 3, 5]. We describe a case of MSGL of the cheek mimicking a venous malformation, treated by diode laser photocoagulation and contextual laser excision.

## Case presentation

**Figure 1:**
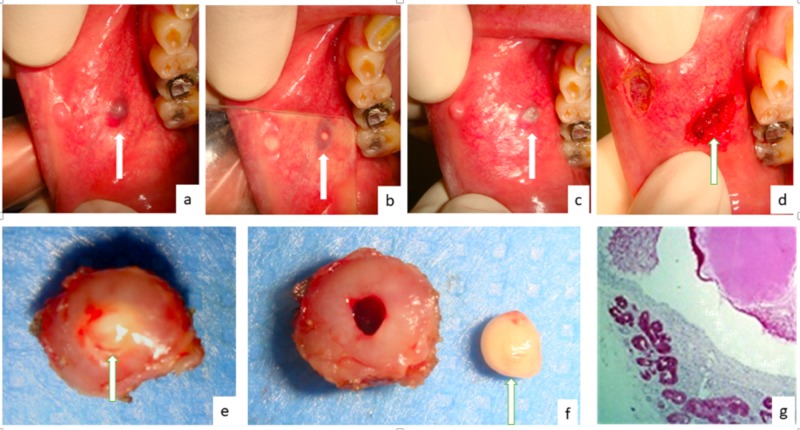
Case presentation a) blue-violet lesions of the cheek (arrow), associated with a small and normally colored lesion appearing as a venous malformation of the cheek; b) under the pressure of transparent glass, a yellowish and hard nodule is detectable within the blue-violet lesion (arrow); c) color variation of white-grey after diode laser treatment (arrow); d) subsequent laser excision of both lesions; e) the entire surgical sample; f) sample with the sialolith removed; g) histological examination showing a cyst-like lumen containing mucina and obstructed by the sialolith.

A 55-year-old male was sent to our observation for a persistent lesion of the cheek. The intraoral examination revealed a firm, nodular lesion with a soft consistency and blue-violet appearance. Under pressure with transparent glass, a hard structure was clearly visible within the lesion. To prevent intraoperative bleeding, the lesion was firstly photocoagulated by diode laser (wavelength 800 ± 10 nm; continuous wave, output energy 5W) and immediately removed by the same laser with different settings (continuous wave, output energy 1W). Bleeding was absent during and after excision and stitches were unnecessary. The histological examination led to the diagnosis of MSGL. The patient was followed-up after seven days and completely recovered after 15 days (Figure 1).

## Discussion

MSGL is frequently misdiagnosed because of unspecific clinical signs and symptoms but also because of resemblance with other benign diseases of the minor salivary gland, especially mucocele, or traumatic lesions of the buccal mucosa [1, 2, 6, 7]. In the reported case, the clinical appearance of a venous malformation of the buccal mucosa was probably related to chewing trauma, as the small lesion detectable nearby, histologically diagnosed as a traumatic fibroma, confirms that. The management of venous malformation by a diode laser, providing a targeted selectivity for oxyhemoglobin, induction of photothermolysis and erythrocyte micro-agglutination and vessel obliteration, is widely reported in the literature [8-10]. Overall, the advantages of diode laser use in several oral surgery and periodontal procedures (e.g., periodontal decontamination, not surgical treatment of gingival overgrowth, surgical excisions of benign oral mucosal lesions and also surgical resection of oral carcinomas) are antibacterial activity, absence of bleeding during cutting with reduction of postoperative edema, unnecessary stitches, and fast mucosal healing [8, 11-16]. Based on this, in the surgical planning of the current case authors decided preliminary treat the vascular component and subsequently remove the entire lesion. 

## Conclusions

Nowadays, among all lasers with surgical capabilities, the diode laser is the most used for the surgical excision of oral mucosa lesions as well as photocoagulation of small and large venous malformations in the head and neck. It is safe, decisive and allows to simplify the procedures for both clinicians and patients.
